# Pervasive Natural Selection in the *Drosophila* Genome?

**DOI:** 10.1371/journal.pgen.1000495

**Published:** 2009-06-05

**Authors:** Guy Sella, Dmitri A. Petrov, Molly Przeworski, Peter Andolfatto

**Affiliations:** 1 Department of Evolution, Systematics and Ecology, The Hebrew University, Jerusalem, Israel; 2 Department of Biology, Stanford University, Stanford, California, United States of America; 3 Department of Human Genetics, University of Chicago, Chicago, Illinois, United States of America; 4 Department of Ecology and Evolution, University of Chicago, Chicago, Illinois, United States of America; 5 Department of Ecology and Evolutionary Biology, Princeton University, Princeton, New Jersey, United States of America; 6 Lewis-Sigler Institute for Integrative Genomics, Princeton University, Princeton, New Jersey, United States of America; University of Arizona, United States of America

## Abstract

Over the past four decades, the predominant view of molecular evolution saw little connection between natural selection and genome evolution, assuming that the functionally constrained fraction of the genome is relatively small and that adaptation is sufficiently infrequent to play little role in shaping patterns of variation within and even between species. Recent evidence from *Drosophila*, reviewed here, suggests that this view may be invalid. Analyses of genetic variation within and between species reveal that much of the *Drosophila* genome is under purifying selection, and thus of functional importance, and that a large fraction of coding and noncoding differences between species are adaptive. The findings further indicate that, in *Drosophila*, adaptations may be both common and strong enough that the fate of neutral mutations depends on their chance linkage to adaptive mutations as much as on the vagaries of genetic drift. The emerging evidence has implications for a wide variety of fields, from conservation genetics to bioinformatics, and presents challenges to modelers and experimentalists alike.

## Introduction

We have known for over half a century that the genome encodes the heritable phenotypes of an organism and that this genetic information is maintained and modified by natural selection on randomly arising mutations. We have learned much in this time about the way in which phenotypes are encoded in the genome. Yet we still know remarkably little about the genetic basis of phenotypic evolution or about how the selective pressures on phenotypes are reflected in genome evolution. Notably, how many sites in the genome encode functions that are maintained by natural selection? How many changes underlie adaptations and how often do such adaptive changes occur? Are adaptive changes clustered in genomic regions associated with particular functions or even in particular genes or are they dispersed throughout the genome? Do adaptive changes tend to occur in coding regions or in regulatory elements? Do most adaptive changes have substantial effects on the fitness of the organism or represent mere “fine tunings?”

Answers to these questions are difficult to garner directly. Although considerable progress has been made in mapping functional regions of eukaryotic genomes, the annotations remain incomplete, and translating the results of biochemical experiments aimed at annotation into statements about fitness effects is not straightforward (e.g., [1]). In turn, direct measurements of the selective effects of mutations are limited in the size of the effect that they can detect and by the specific environmental conditions of the assay (reviewed in [2]). And while the genetic basis of several relatively simple adaptations have recently been elucidated (e.g., [3]–[9]), these studies do not address questions about the extent or typical strength of positive selection.

In principle, patterns of variation within and between species can provide answers to these questions, as well as help characterize the intensity and rate of adaptation. Polymorphism within species and divergence between species carry the footprints of evolutionary events, including those of natural selection, and can therefore be used to learn about how natural selection acts on organisms and how this process shapes genomes. To interpret these footprints of selection, however, we need to know what type of mutations occur spontaneously and at what rates, and to have a model for how the varying fitness effects of these mutations become reflected in the observed patterns of polymorphism and divergence.

The consequences of newly arising mutations in the genome can be classified as **neutral**, if they have no or almost no effect on fitness, **deleterious**, if they have a pronounced negative effect on fitness, and **advantageous**, if they have a significant beneficial fitness effect. This classification admittedly ignores many known phenomena, such as epistatic interactions among mutations, frequency-dependent selection, heterozygous advantage, and spatially and temporarily varying selective pressures within species [10],[11]—all of which can substantially affect fates of mutations in populations. However, this rough classification is illustrative and serves as a useful starting point in thinking about footprints of evolutionary events in the genome.

Whereas the distribution of selective effects remains largely unknown [2], intuition as well as experimental and evolutionary analyses suggest that there are many more deleterious and neutral mutations than there are advantageous ones [2],[12]. Consider an analogy between the genome of an organism and the blueprint of a radio. Introducing random changes into the blueprint is much more likely to disrupt one of the radio's systems, or to not affect its functionality in a noticeable way, than to improve it. By analogy, random mutations in the genome are more likely to be deleterious if they arise in a functionally important segment of the genome, or neutral, if they occur in a region of the genome that is devoid of functional importance, than they are to be advantageous to the organism.

The differing fitness effects of mutations shape their contribution to genetic variation within and between species. Although newly arising mutations with strong deleterious effects may be common, they will very rarely rise to substantial frequencies in the population, let alone reach fixation, because they are efficiently purged by natural (purifying) selection. Thus, they should be observed only rarely in polymorphism and almost never in divergence [12]. In contrast, beneficial alleles may contribute substantially to divergence: even though they occur infrequently, their probability of fixation can be orders of magnitude greater than that of neutral or deleterious mutations [13]. Beneficial alleles may also contribute to polymorphism, but to a much lesser degree: not only are they rare among new mutations, but even those adaptive mutations that are destined for fixation—and thus traverse the range from low to high population frequencies—do so rapidly, decreasing the chance of their being sampled while polymorphic. Instead, most variation observed within a species is likely to be neutral, both because many new mutations may be neutral and because those neutral alleles that rise to substantial frequencies by chance will tend to persist for a relatively long time before they are lost or fixed. Many differences between species may also be neutral, if the fraction of newly arising neutral mutations is large enough to offset their low chance of fixation. These considerations therefore suggest that newly arising mutations tend to be deleterious and neutral, that the observed variation within species is predominantly neutral, and that the fixed differences between species are advantageous and neutral.

From this point of view, questions about the role of selection in genome evolution can then be recast as:

1. Precisely what fraction of newly arising mutations is deleterious? In many ways, this is equivalent to asking what fraction of the genome is functionally important.
2. What fraction of the fixed differences between species is advantageous?

As described below, positive and negative selection also impact levels of polymorphism at genetically linked neutral sites, and the magnitude of these effects reflects the extent and intensity of natural selection. Therefore, a third, related question is:

1. To what extent is the observed neutral genetic variation within species shaped by linkage to selected alleles?

The Neutral Theory [12]—the dominant view of genome evolution for the last four decades—can be presented in terms of its answers to these three questions. It states that: (i) The vast majority of newly arising mutations are neutral or strongly deleterious. (ii) Most fixed differences between species are neutral, with a negligible contribution of adaptive mutations. (iii) The effects of both positive and negative selection at linked loci on the dynamics of neutral alleles can be ignored. Thus, the Neutral Theory postulates not only that the vast majority of the variation within and between species is neutral, but also that the changes in population frequencies of neutral alleles are not affected by selection but instead are fully governed by random genetic drift—the dynamics that result from the random sampling of alleles across generations. The nearly neutral extension of the Neutral Theory [14] shares these assumptions with one modification: it postulates that polymorphism and divergence at functionally important sites is predominantly nearly, rather than strictly, neutral. The nearly neutral range of selective effects is defined as the range where the effects of genetic drift are comparable to those of natural selection (i.e., 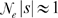, where *N_e_* is the effective population size and *s* the selection coefficient), such that deleterious mutations may still rise to substantial population frequencies by chance [12],[14]. While the neutral and nearly neutral view of molecular evolution have not gone uncontested (e.g., [10], [15]–[17]), these theories have formed the basis of theory and inference in evolutionary genomics over the past four decades [18]–[20] and increasingly in other fields, from bioinformatics to conservation biology.

Recent evidence, however, is calling these assumptions into question. While the studies have been conducted in a range of taxa, the strongest case comes from *Drosophila*, where multiple lines of inquiry challenge the basic tenets of the Neutral Theory. We therefore focus on the evidence from this taxon. We describe results suggesting that a hitherto unsuspected fraction of the *Drosophila* genome is involved in function and that adaptive changes in *Drosophila* are frequent, widespread, and possibly often of substantial selective effect. On this basis, we argue that positive selection cannot be ignored in the study of genome evolution in this taxon, even when truly neutral changes in nonfunctional regions are considered. As we discuss below, these findings cast doubt on the validity of the Neutral Theory in *Drosophila*, and possibly in other species, raising new and challenging questions for experimentalists and theoreticians alike.

## Evidence for Widespread Purifying and Positive Selection

The fraction of deleterious alleles among newly arising mutations and the fraction of between-species differences that are adaptive can be estimated from sequence data by extending a framework first developed by McDonald and Kreitman [21]–[25] (see Box 1).

By applying this methodology to polymorphism data from *D. melanogaster*, the fraction of deleterious newly arising mutations was estimated to be ∼94% at amino acid sites, ∼81% in untranslated regions (UTRs), ∼56% in introns, and ∼61% in intergenic regions [26] (see Table 1). While the conclusion that the vast majority of amino acid mutations are under purifying selection is not surprising [12], the finding that close to two-thirds of mutations in noncoding regions are also deleterious marks a profound shift in our view of the extent of natural selection in the *Drosophila* genome. Because purifying selection in a genomic region is the evolutionary hallmark of its importance to the organism, these findings suggest that most of the euchromatic portion of the *Drosophila* genome is functionally important [26]–[28].

**Table 1:** The fraction of neutral mutations and adaptive divergence estimated from diversity and divergence in *D. melanogaster*.

Site Class	Sub-Parameter	% of genome	diversity (π)	divergence (*K*)	*f* (π/π_0_)	α (Equation. 4)
Coding	Synonymous	4.5%	2.9%	13.6%	—	—
	Nonsynonymous	14%	0.2%	1.7%	0.06	0.50
Noncoding	UTRs	6.0%	0.5%	4.5%	0.19	0.44
	Introns (<100 bp)	2.9%	—	—	—	—
	Introns (>100 bp)	55%	1.3%	6.7%	0.44	0.12
	Intergenic	18%	1.0%	5.7%	0.34	0.18

Average pairwise diversity (π) and divergence (*K*) per site are from [26]. An estimate of the fraction of neutral mutations, *f*, was obtained from equation 3, assuming that the expected neutral diversity, π_0_, is equal to the average π at synonymous sites. An estimate of the fraction of adaptive divergence, α, was obtained from equation 4 and averages of π and *K* across loci.

Estimates of adaptive substitution rates in *Drosophila* are posing an even greater challenge to the dominant view. Numerous studies have estimated that 40–50% of the amino acid substitutions in *Drosophila* species are adaptive (see Table 1 for an example with data from [26]). These estimates are derived from a variety of statistical methodologies and datasets from several *Drosophila* species, including *D. melanogaster* and *D. simulans* [24], [26], [29]–[37], *D. virilis* and *D. americana* [38], and *D. miranda* and *D. pseudoobscura* [39]–[41]. Moreover, this proportion appears to be fairly uniform across genes, suggesting that adaptive evolution in *Drosophila* is not clustered in particular subsets of genes ([31],[32],[36], although see [29],[42],[43]). In turn, approximately one of five substitutions in noncoding regions appears to be adaptive, with estimates of beneficial substitutions rates in UTRs reaching 34–70% [26],[35],[37],[44]. Together, these estimates indicate that *Drosophila* species experience an adaptive amino acid substitution every 200–400 generations and one in noncoding regions at potentially more than five times that rate [26]. If reliable, they suggest that the central premise of the Neutral Theory—that adaptations contribute negligibly to divergence between species—is invalid in *Drosophila*.

These conclusions are still tentative, however, due to statistical problems with the estimation procedures and possible departures from the simplifying assumptions of the model on which they rely. While we outline these limitations in terms of nonsynonymous and synonymous sites, they hold more generally. (i) One statistical difficulty is that counts of synonymous polymorphisms per gene, which appear in the denominator in Equations 3 and 4 in Box 1, are usually small and therefore lead to noisy estimates of parameters *f* and α per gene (both because of sampling variance and variation inherent in the evolutionary process). A common solution is to pool sparse counts of polymorphism and divergence across genes; however, pooling can introduce systematic biases into the estimation procedure, in particular when there is a negative correlation between neutral diversity and amino acid divergence levels [24],[34],[36]. (ii) A subset of synonymous mutations is likely to be under weak purifying selection rather than neutral (e.g., [37], [45]–[47]), leading to a reduction in levels of synonymous polymorphism compared to neutral levels and hence to an over-estimate of *f*. Moreover, because purifying selection on synonymous sites reduces divergence more than polymorphism, it can also cause an over-estimate of the fraction of adaptive amino acid substitutions, α [48]. (iii) A non-negligible fraction of nonsynonymous mutations may be weakly rather than strongly selected [26],[29],[37],[40],[49],[50]. These mutations are likely to be predominantly deleterious, leading to under-estimates of *f* and α. Comparisons of the allele frequency spectrum at synonymous and nonsynonymous sites indicate that, on average, weak purifying selection is more pervasive at nonsynonymous sites, suggesting that the overall effects of weak selection should tend to lead to an under-estimate of both *f* and α [26]. The biases due to weak selection can, in principle, be reduced by excluding rare polymorphisms [22],[23],[26],[51],[52] or by using estimation methods that take into account the possibility of weak selection (e.g., [53]). (iv) Perhaps the most problematic assumption underlying McDonald-Kreitman estimates is that the fraction of newly arising mutations that are neutral, *f*, which is estimated from polymorphism data in one species, has remained constant during the evolutionary history of the two species. Several studies have discussed how a nonequilibrium demographic history can invalidate this assumption when selection is weak, potentially resulting in misleading estimates of the rate of adaptive substitutions [21], [23], [29], [54]–[56]. Nonetheless, the estimates of α are consistently high across studies of a variety of *Drosophila* species with different demographic histories (see references above), making it highly unlikely that the findings of pervasive adaptive substitutions are solely attributable to such biases.

## Signatures of Hitchhiking and Background Selection

**Figure 1:**
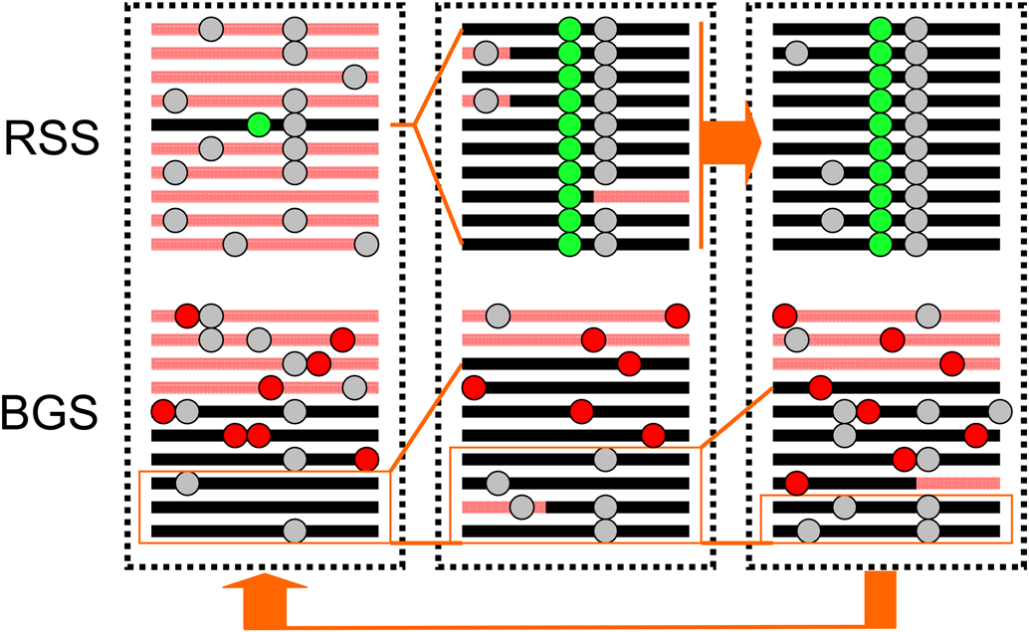
The effect of positive and negative selection on linked neutral sites. This cartoon depicts a population of ten chromosomes, subject to recurrent selective sweeps (RSS) or background selection (BGS). Neutral mutations are shown as gray circles, the beneficial mutation in green, and deleterious mutations in red. RSS: An adaptive mutation destined for fixation arises on a particular haplotype, i.e., linked to a specific combination of neutral alleles at polymorphic sites. As it increases in frequency in the population, so does that genetic background. All pre-existing alleles not on the selected background are lost from the population, unless they recombine onto chromosomes carrying the beneficial allele before fixation. Thus, a “selective sweep” causes a reduction in the level of polymorphism as well as a distortion of allele frequencies in the vicinity of the beneficial substitution [57],[58],[126]. After fixation, diversity will be reintroduced by mutation, but a footprint of the substitution may remain for a long time (up to *N*_e_ generations; [78]). BGS: The balance between a steady flux of deleterious mutations and purifying selection generates a stable partition of chromosomes in a population, depending on how many deleterious mutations they carry. Chromosomes with deleterious mutations will be eliminated relatively quickly from the population by purifying selection, but this class is constantly replenished by new deleterious mutations. In the absence of recombination, a new neutral mutation can remain in the population for a long period of time and rise to high population frequencies only if it appears on a gamete that is free of deleterious mutations, and hence is not destined to be rapidly eliminated. The effect of this “background selection” against deleterious mutations is a reduction in the level of neutral polymorphism [61], as well a downward shift in their population frequencies, because of the relative excess of short-lived (and hence low frequency) neutral mutations [63].

Independent evidence about the role of selection can be garnered by seeking its signature in neutral polymorphism data. An adaptive substitution can markedly affect the dynamics of neutral alleles in its genomic vicinity, leading to lower diversity and a skew in the allele frequency spectrum at linked sites [57],[58] (see Figure 1). These effects decrease with genetic distance between the neutral and selected alleles, as recombination uncouples their dynamics. In turn, the effects increase with the intensity of positive selection, because a more strongly advantageous allele reaches fixation faster, leading to fewer recombination events between the selected and neutral sites during its ascent. Under simplifying assumptions, the beneficial substitution of a single allele can influence patterns of neutral polymorphism within a region of length ∼0.1*s*/*r*, where *s* is the beneficial selection coefficient and *r* is the recombination rate per base pair (bp) [13],[57],[59]; as an illustration, for a selection coefficient of 1%, as much as 100 kb could be affected in regions of average recombination in *Drosophila*. Thus, if adaptations are indeed as frequent as the McDonald-Kreitman-based estimates suggest and a substantial fraction of these adaptations are driven by sufficiently strong positive selection, the Neutral Theory's assumption of a negligible effect of positive selection on the dynamics of neutral and weakly selected alleles within species may prove erroneous [60].

In addition to adaptation, “background selection” against deleterious mutations can also affect the dynamics of linked neutral alleles [61], leading to lower diversity and a skew toward rare alleles (see Figure 1). The magnitude of the effects on diversity and the allele frequencies increase with the rate of deleterious mutation and decrease with the recombination rate, because recombination allows neutral mutations to escape onto chromosomes carrying fewer deleterious mutations [62]. The importance of these effects also varies with the intensity of purifying selection. The maximal effect on polymorphism levels is for intermediate selective effects, because strongly deleterious alleles are eliminated from the population too quickly to be associated with many neutral alleles, and weakly deleterious ones are eliminated too slowly to remove much neutral variation [63]. In contrast, the shift toward lower population frequencies increases as the intensity of purifying selection decreases and becomes detectable only for weak deleterious selection, when the overall reduction in polymorphism is minimal [63],[64].

## The Relationship between Diversity and Recombination

**Figure 2:**
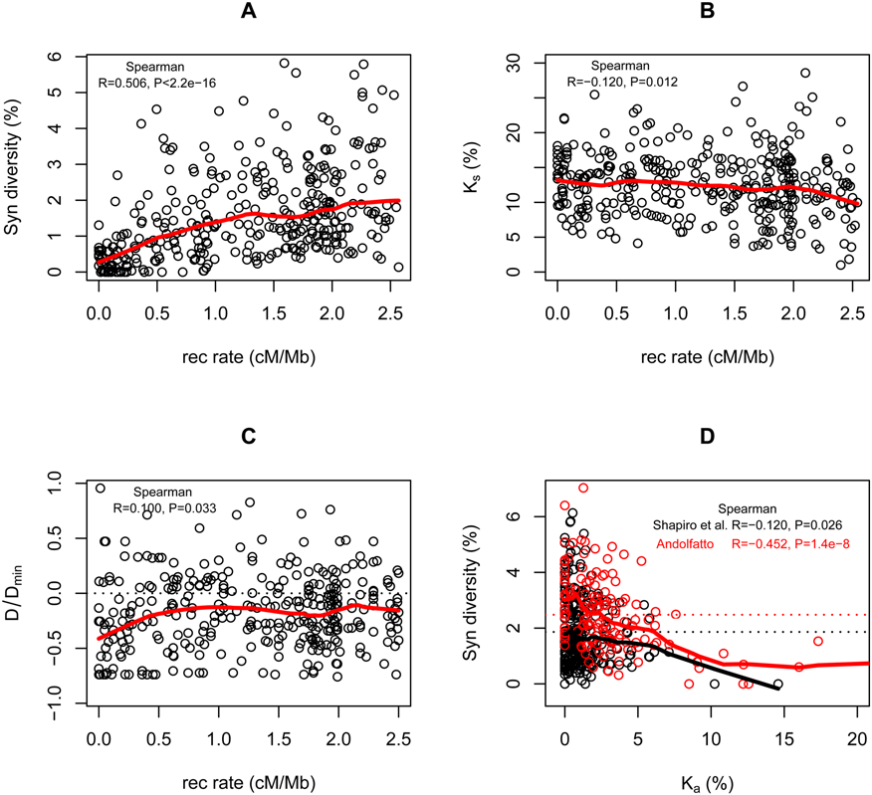
Correlations in polymorphism data from *D. melanogaster*. (A) Levels of synonymous site diversity versus recombination rates. The effects of the rate of amino acid divergence (*K_a_*) and the rate of synonymous site divergence (*K_s_*) have been controlled for by partial regression, with negative values set to zero. (B) *K_s_* versus recombination rates. The effect of *K_a_* has been controlled for by partial regression, with negative values set to zero. (C) A summary of the allele frequency spectrum at synonymous sites versus recombination rates; more negative values of the statistic reflect a higher proportion of rare alleles. The numerator is Tajima's *D* [127] and the denominator is the minimum value *D* (in absolute value) can take given the sample size and number of segregating sites [128]. (A–C) are based on the polymorphism data of Shapiro et al. [34], and recombination rates estimated by Comeron et al. [129]. For the Shapiro et al. data, 349 loci with >50 synonymous sites were used and only African individuals are included. (D) Levels of synonymous site diversity as a function of *K_a_*. In red are the 137 X-linked loci surveyed by Andolfatto [33]. In black are autosomal loci surveyed by Shapiro et al. [34]. For both data sets, the effect of *K_s_* has been controlled for by partial regression, with negative values set to zero. For the Shapiro et al. data, 265 loci with recombination rates >0.5 cM/Mb and >50 synonymous sites were included. The red and black dotted lines represent average levels of synonymous π in the Andolfatto and Shapiro et al. datasets, respectively. Thick red and black lines indicate Lowess fits to the data. All *p*-values are one-tailed.

The effects of selection on the dynamics of neutral and weakly selected alleles can be sought by comparing patterns of polymorphism and divergence across recombination environments. If deleterious mutations and adaptive substitutions occur at similar rates throughout the genome, their effects on neutral polymorphism should be greater in regions with lower recombination, where a neutral allele is linked to a larger number of selected sites. In accordance with this expectation, polymorphism is markedly reduced toward centromeres and telomeres, and on the Y chromosome and Chromosome 4 of *D. melanogaster* and *D. simulans*, genomic regions known to experience reduced levels of crossing-over [34], [35], [65]–[71]. These observations cannot be explained entirely by mutagenic effects of recombination, because neutral divergence levels are not markedly lower in regions of low crossing-over [35],[67],[72] (Figure 2B). More generally, levels of polymorphism increase with estimated crossing-over rates in *D. melanogaster* (Figure 2A), *D. simulans* [35], and *D. pseudoobscura* [34],[35],[67],[71],[72]. Whereas in the *D. melanogaster* group divergence levels appear to correlate too weakly with crossing-over rates to account for this correlation, a firm conclusion awaits higher-resolution genetic maps in these species [35],[67],[72]. In addition, allele frequencies at synonymous sites are skewed towards rare alleles, with a slightly more pronounced skew in regions of low recombination [34],[71] (Figure 2C). Both observations about polymorphism levels and allele frequencies provide strong support for the influence of natural selection on linked neutral and weakly selected alleles.

Distinguishing the relative contributions of selective sweeps and background purifying selection to the correlations, however, has proven difficult [72]–[76]. Models of recurrent selective sweeps can explain both a reduction in diversity and a skew toward lower frequencies seen in regions of reduced recombination (e.g., [58],[76]). In turn, background selection caused by strong purifying selection can account for the reduction in polymorphism but not the skew [69],[71], whereas background selection caused by weakly deleterious mutations can account for the skew but not the reduction [64]. Whether the observed correlations can be explained by one or both models awaits further theoretical work and a better characterization of the distribution of the fitness effects of both beneficial and deleterious mutations.

## The Relationship between Diversity and Amino Acid Divergence

**Figure 3:**
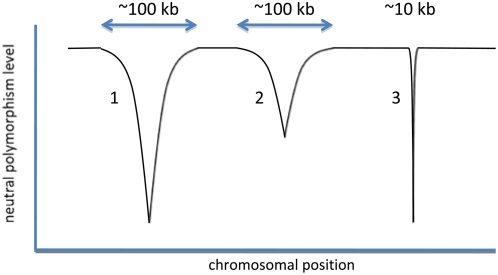
Cartoon of the effects of recurrent selective sweeps on patterns of genetic variation along the genome. In this cartoon, several beneficial substitutions have occurred within this region, reducing levels of diversity relative to background levels. The sweep labeled 1 was driven by strong selection and occurred very recently, leading to a sharp decrease in diversity at linked sites. Sweep 2 was associated with a similarly strong selective coefficient, but occurred further in the past, such that levels of polymorphism surrounding the site have had some time to recover through mutation and random genetic drift. Sweep 3 occurred recently, but was associated with a weaker selective coefficient, thereby reducing polymorphism in a smaller region. We emphasize that, in practice, diversity patterns alone are likely to be an unreliable indicator of selective sweeps, as there are numerous other sources of heterogeneity.

The relationship between neutral diversity and divergence at functional sites can be particularly informative about the effects of positive selection on neutral and weakly selected alleles. Figure 3 illustrates the effect of recurrent selective sweeps on levels of neutral polymorphism along a genomic region, assuming a uniform recombination rate. As shown in this cartoon, the spatial pattern of neutral polymorphism at a given point in time, i.e., the number and width of troughs in neutral polymorphism levels, carries information about the frequency and intensity of adaptations. In practice, however, heterogeneity in polymorphism alone may be an unreliable indicator of selective sweeps, because other evolutionary forces, notably demographic processes and heterogeneity in mutation rates, can also produce spatial heterogeneity in levels of neutral variation (e.g., [77]–[81]).

Considering polymorphism data in conjunction with divergence data can reduce the confounding effects of other evolutionary processes. Specifically, because adaptive substitutions that cause selective sweeps will appear as divergence at functional sites, recurrent selective sweeps are expected to generate a negative correlation between levels of neutral polymorphism and levels of divergence at functionally important sites. In addition, the spatial scale over which these correlations are observed may be informative about the parameters of adaptive substitutions.

This reasoning motivated two recent studies. Andolfatto [33] examined the relationship of synonymous polymorphism in *D. melanogaster* to the rate of protein evolution between *D. melanogaster* and *D. simulans* among a set of X-linked genes in highly recombining regions. He detected a negative correlation between levels of synonymous polymorphism and the rate of amino acid evolution (Figure 2D), which is not driven solely by few rapidly evolving genes [33]. In a concurrent study, Macpherson et al. [82] examined the relationship between synonymous polymorphism in *D. simulans* and amino acid divergence between *D. melanogaster* and *D. simulans*, in 100-kb windows, a scale that is an order of magnitude greater than that of a typical gene. Focusing on all highly recombining regions of autosomes, they found that levels of polymorphism are negatively correlated with the number of amino acid substitutions. Because recent selective sweeps are expected to produce sharp dips in levels of polymorphism (Figure 3), regions with frequent adaptations should exhibit not only reduced levels of diversity but also greater contrasts between minimal and background levels of polymorphism (i.e., greater heterogeneity in diversity levels). To test this prediction, they examined the relationship between the ratio of minimal to average synonymous polymorphism, *Q_S_*, and amino acid divergence, in 100-kb windows. They found a strong negative correlation, with a consistent decrease in *Q_S_* throughout the range of amino acid divergence, a finding that further supports the prevalence of selective sweeps.

While both papers reported a significant negative correlation between levels of neutral polymorphism and amino acid divergence, the scale of measurement differed greatly—from single genes [33] to 100-kb windows [82]—raising the question of whether the larger-scale finding arises from an underlying correlation at a smaller scale. Assessing this question by permutation, Macpherson et al. concluded that the correlation at 100-kb scales is due to effects that operate at distances substantially beyond than that of a gene [82]. A possible interpretation is that the correlation on a genic scale primarily reflects the signature of weak sweeps, while those on the 100-kb scale mostly reflects the effects of strong sweeps. Since a weakly beneficial substitution only causes a reduction in diversity levels nearby, both the substitution and the reduction are likely to be observed in the same gene. In turn, the 100-kb scale may be large enough to include both a strongly beneficial substitution and the reduction in diversity that it caused, but may be too large for the effects of weakly beneficial substitutions to be detected. If this interpretation is correct, then the signatures of selective sweeps on different spatial scales may carry valuable information about the distribution of adaptive selective effects.

Although these recent results provide evidence for the effects of recurrent selective sweeps on neutral polymorphism even in high recombination regions of the *Drosophila* genome, the specific observations still await a unifying interpretation. Among open questions is the extent to which background selection contributes to these patterns. For example, can background selection account for the negative correlations between amino acid divergence and polymorphism in regions of high crossing-over? On the one hand, genes with many amino acid sites under purifying selection experience more background selection, leading to lower neutral diversity where there is a lower substitution rate (i.e., the opposite of what is observed). On the other hand, background selection could also reduce the efficacy of selection against weakly deleterious amino acid mutations, leading to a higher rate of amino acid substitution. Even less clear is whether background selection can explain the greater heterogeneity in polymorphism observed in regions with elevated amino acid divergence. To answer these questions, we need a better understanding of the way background selection shapes spatial patterns of neutral polymorphism [83], and a more accurate characterization of the selective parameters and spatial distribution of deleterious mutations, as well as better genetic maps for *Drosophila*.

## Inferring the Rate and Strength of Adaptation at the Genomic Level

The relationships of polymorphism with recombination rates and with amino acid divergence can be used to infer the rate and strength of adaptations (for estimates of deleterious selection parameters, see [2],[25],[84]). Such inferences can provide estimates of the rate of adaptation that are independent of those of the McDonald-Kreitman approach, because the methodologies rely on different signatures of the adaptive process. In addition, they yield estimates of the selective effect of beneficial substitutions (e.g., addressing whether they are typically large or not), which are not accessible using a McDonald-Kreitman-based approach.

The first approach was developed by Wiehe and Stephan [85], who used the relationship between levels of synonymous polymorphism and recombination rates in *D. melanogaster* to infer the product of the rate and strength of adaptive substitutions. To this end, they derived a formula for the expected heterozygosity, π, under a model of recurrent selective sweeps in a random-mating population of constant size:
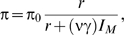
(1)where π_0_ is the expected heterozygosity in the absence of selective sweeps, *r* is the recombination rate per bp, ν is the rate of adaptive substitution per bp, γ = 2*Ns* where *s* is the adaptive selective advantage, *N* is the population size, and *I_M_*≈0.075. They then found the values of π_0_ and νγ that lead to the best fit to the observed relationship between π and *r*. This estimation procedure assumes that the rate and strength of positive selection are the same across the genome and therefore that differences in polymorphism levels among regions reflect only the effects of selective sweeps in varying recombination environments. Wiehe and Stephan arrive at a compound estimate of νγ greater than 1.3×10^−8^ (see Table 2), which implies a mean reduction in polymorphism of 50% in regions of low recombination (where *r*≈0.1 cM/Mb) and of 4% in high recombination regions (*r*>2.5 cM/Mb) (see also [68],[86]).

**Table 2:** Estimates of selection parameters in *Drosophila*.

Reference	Dataset	*s*	ν	νγ
Wiehe and Stephan 1992 [85]	17 X-linked and autosomal genic regions in *D. melanogaster*	—	—	>1.3×10^−8^
Li and Stephan 2006^a^ [88]	∼200 X-linked, noncoding regions in *D. melanogaster* (average 512 bp)	0.2–0.5%	6–9×10^−11^	—
Andolfatto 2007 [33]	137 X-linked gene coding fragments in *D. melanogaster* from regions of high recombination (700–800 bp)	∼10^−5^	7.5×10^−10^	3×10^−8^
Macpherson et al. 2007 [82]	100-kb windows for all autosomal regions of high recombination in D. simulans	1%	3.6×10^−12^	10^−7^
Jensen et al. 2008^b^ [92]	Same as Andolfatto 2007	0.2%	4×10^−11^	4×10^−7^

We note that these estimates are not really comparable, as they are derived under different assumptions, not to mention different species and modes of inheritance.

In the column titled *s* is the reported estimate of the strength of selection, under ν the reported estimate of the rate of adaptive substitutions per base pair per generation and under νγ = 2*N_e_s*νis the reported compound estimate (see text).

a Two populations are used for inference, resulting in two separate estimates of the parameters.

b The parameters were estimated from the mode of the posterior distribution sample, assuming specific distributions for the selection coefficient and rates of adaptation.

With the above approach, the rate and strength of recurrent selective sweeps appear as a compound parameter (νγ), because doubling the rate (and thus the number) of selective sweeps that affect a neutral site is equivalent in its effects on mean diversity to doubling the intensity (and thus the distance) over which sweeps have an effect. Thus, Wiehe and Stephan were not able to distinguish between the rate (ν) and the strength of selection (*s*). Recently, several attempts have been made to estimate these parameters separately, using information about the rate of adaptive divergence provided by the McDonald-Kreitman based estimates. For example, Eyre-Walker [87] calculated that, given Wiehe and Stephan's estimate of νγ above and estimates of adaptive divergence in proteins and non-coding DNA, γ lies in the range 350 to 7,000 (i.e., 10^−4^<*s*<2×10^−3^, assuming *N_e_*∼2×10^6^ [33]).

Andolfatto [33] used a similar approach to estimate the rate (ν) and intensity of adaptations (*s*), but instead relied on the relationship between levels of synonymous polymorphism and rates of protein evolution. He assumed that the rate of adaptation is proportional to the rate of protein evolution ν = α*K_a_*. Substituting this relation into Equation 1 yields a relationship between expected levels of neutral polymorphism and rates of protein evolution
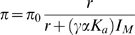
(2)that can be used to infer the compound parameter γα; multiplying this parameter by the average rate of protein evolution 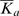 provides an estimate of the rate and strength compound parameter (i.e., of 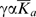). Application of a maximum likelihood method that accounts for both the mean and variability in polymorphism levels across genes under recurrent selective sweeps yields 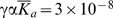, which is within an order of magnitude of other estimates [85]–[89] (see Table 2) and implies a ∼15% reduction in neutral diversity levels on average in high recombination regions (*r*>2 cM/Mb). To obtain the intensity of selection, *s*, from this compound estimate, Andolfatto [33] inferred α using the McDonald-Kreitman-based approach [32]. From this, he estimated that ν is approximately 7.5×10^−10^ per generation per bp in protein coding regions (i.e., that there was one adaptive substitution every ∼200 generations) and that γ≈40 (i.e., *s*≈10^−5^)—very weak selection that is only slightly above the nearly neutral range.

**Box 1. Estimating levels of constraints and rates of adaptation in proteins**

Consider two distinct classes of mutations found in protein-coding genes: nonsynonymous mutations that change the amino acid and synonymous mutations that alter the codon but not the amino acid. Assume that nonsynonymous mutations can be either strongly deleterious or neutral and that synonymous mutations are neutral. Under this model, neutral nonsynonymous and synonymous mutations contribute similarly to the polymorphism, whereas deleterious nonsynonymous mutations contribute negligibly. Thus, the ratio of nonsynonymous to synonymous polymorphism reflects the fraction of new mutations that are neutral, *f*, while the fraction of deleterious nonsynonymous mutations is given by 1 – *f*. In practice, levels of polymorphism per nonsynonymous site, π*_a_*, and per synonymous-site, π*_s_*, are calculated in a population sample of DNA sequences in coding regions and *f* can be estimated as:
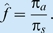
(3)

In turn, the fraction of adaptive fixed differences between species, α, can be estimated from the number of substitutions per nonsynonymous site, *K_a_*, and the number of substitutions per synonymous site, *K_s_*. If there were no adaptive amino acid substitutions, such that all the non-synonymous and synonymous polymorphism and divergence were generated by neutral mutations, we would expect that:
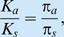
because neutral mutations would contribute in similar proportion to polymorphism and divergence at nonsynonymous and synonymous sites. By the same token, if a fraction 1 – α of amino acid substitutions is neutral and a fraction α is adaptive, then we expect that 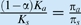. Therefore, the fraction of amino acid divergence that is adaptive can be estimated as [24]:
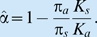
(4)

While this explanation focused on amino acid sites and relied on synonymous mutations as a neutral reference, similar estimates can in principle be obtained from a comparison of any two sets of sites, one of which is putatively evolving neutrally.

These parameters can also be estimated using a different approach: while the mean diversity depends on amino acid divergence only through the compound parameter νγ, the heterogeneity in diversity levels (e.g., summarized by the statistic *Q_S_* [82]) depends differently on the rate and the strength of recurrent selective sweeps, thereby allowing these two parameters to be estimated separately [82]. Independently of the strength of selection, the minimum diversity will occur around the last beneficial substitution. In turn, the level of diversity at that position will depend solely on how recently the last advantageous allele fixed, i.e., on the rate of adaptive substitutions. Based on these considerations, Macpherson et al. [82] inferred the rate and strength of recurrent selective sweeps in *D. simulans* by simultaneously fitting a model to the relationship of divergence to mean polymorphism levels and *Q_S_*. Their estimate of the rate of adaptive substitutions is ∼3.6×10^−12^ gen^−1^ bp^−1^, or approximately 1 every 3,000 generations (Table 2). Although an order of magnitude lower than Andolfatto's estimate of ν based on the McDonald-Kreitman methodology, this estimate again suggests the occurrence of frequent adaptations in *Drosophila*. Macpherson et al.'s estimate of selective intensity, however, is *s*≈1%, corresponding to strong selection (i.e., orders of magnitude above the nearly neutral range), while the compound parameter that derives from these estimates, 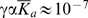, is within an order of magnitude of those obtained in *D. melanogaster*.

The differences among estimates of selection intensity and the rate of adaptation (but not the compound parameter) are striking. How could they be explained? Obviously, they could arise, at least in part, from the use of different (although closely related) *Drosophila* species and loci with different modes of inheritance (i.e., sex-linked versus autosomal). However, other factors may also be important. First, the spatial scale over which the relationships are examined may influence the estimates: for example, Andolfatto considered data at the genic scale and obtained an estimate of *s* that would lead to a reduction over approximately 500 bp (i.e., 0.1*s*/*r*)—the scale considered—while Macpherson et al. focused on 100-kb windows and found an estimate of the strength of selection that would lead to a sweep over ∼40,000 bp—again the scale considered. Second, if the majority of adaptive substitutions are driven by weak selection and a minority is driven by strong selection, polymorphism patterns may primarily reflect the minority of stronger sweeps while the McDonald-Kreitman based estimates should reflect both. This reasoning may explain why Macpherson et al., who rely on the signature of sweeps in polymorphism data, infer a rate of adaptation that is considerably lower than the McDonald-Kreitman-based estimates and, for those adaptations, a higher intensity of selection. Indeed, a back of the envelope calculation indicates that the results from the two studies can be reconciled if ∼95% of amino acid adaptive substitutions are driven by weak selection and ∼5% by strong selection.

An important limitation of all these inference methods is their reliance on the over-simplified demographic assumptions of a panmictic population of constant size. Although demographic processes, such as the population bottlenecks and expansions that are known to have occurred in *Drosophila* species [81],[90],[91], are unlikely to single-handedly generate the relationship between polymorphism levels and recombination or functional divergence, they play a role in shaping patterns of neutral polymorphism and thus will likely modify these relationships. To address this shortcoming, Li and Stephan [89] used information about the frequency spectrum across noncoding loci to infer a demographic model for European and African populations of *D. melanogaster*. They then estimated the number and intensity of beneficial substitutions that have occurred in both populations based on deviations of the frequency spectrum from the neutral expectation, under the inferred demographic model. This approach yielded an adaptive rate of ∼6×10^−11^ gen^−1^ bp^−1^ and an intensity *s*≈0.2% in the African population, and a rate of ∼9×10^−11^ gen^−1^ bp^−1^ and *s*≈0.5% in the European populations, assuming no migration between European and African populations since they split. While this approach has the attractive feature of accounting explicitly for plausible demographic effects, its reliance on polymorphism data alone (rather than on the relationship to functional divergence or recombination) may render the estimates quite sensitive to misspecification of the demographic model, as well as to additional sources of heterogeneity in diversity patterns [92].

Future inference methods would therefore gain from combining the strengths of existing approaches: incorporating information about recombination and functional divergence, which more distinctively capture the effects of natural selection on diversity, while being relatively robust to uncertainty about demographic history or incorporating its effects explicitly. Methods would further benefit from explicitly using information from different spatial scales, and, in turn, allowing for variation in selection coefficients rather than assuming a single value (as done by Jensen et al. [92]). Another complication that should be addressed is that, in theory, background selection could also contribute to an association between neutral polymorphism and recombination or functional divergence, a contribution that could be more substantial when combined with nonequilibrium demographic processes (for example, if the effects of a population bottleneck on diversity levels are proportionally greater in genomic regions with more background selection).

## Implications for the Neutral Theory in *Drosophila*

The analysis of nucleotide variation data within and between *Drosophila* species provides tentative answers to the three questions posed in the Introduction, suggesting that: (i) most of the genome is under purifying selection and (ii) a large fraction of divergence at amino acid, and possibly in noncoding regions, is beneficial. This answer is provided by both the McDonald-Kreitman-based estimates and by the relationships between diversity and recombination and between diversity and functional divergence, patterns that are most readily explained by recurrent selective sweeps. (iii) The dynamics of neutral and weakly selected alleles are affected substantially by selection at linked sites and, in particular, by recurrent selective sweeps. Because the Neutral Theory assumes a negligible contribution of adaptive substitutions to divergence and a negligible effect of selection on neutral or weakly selected polymorphism, its validity as a depiction of the processes of molecular evolution in *Drosophila* is now in question.

## How Do These Findings Change Our View of Molecular Evolution?

By undermining the tenets of the Neutral Theory, these findings have numerous implications for our interpretation of genetic variation. For example, the extent of sequence conservation between species is widely used to measure the density of functionally important sites (sometimes referred to as functional constraint), with the implicit assumption that changes are either neutral or deleterious (e.g., [20],[28]). But if adaptive substitutions are as common as the McDonald-Kreitman-based estimates suggest, then divergence reflects similar contributions of both neutral and adaptive changes. And since adaptive changes are clearly of functional importance, equating functional importance with sequence conservation could be misleading. Similarly, the comparison of selective pressures using *K_a_*/*K_s_* ratios conflates the contributions of adaptive and neutral changes to sequence divergence; a high ratio could reflect little constraint, or a combination of adaptation and purifying selection. In other words, if adaptations are common, then characterizing selective pressures across species or among genomic regions requires approaches that explicitly allow for positive, negative, and neutral changes (in terms of parameters such as *f* and α) rather than combining their effects into a single parameter, as done in many widely used methods. The McDonald-Kreitman methodology offers one such alternative—one that, with the availability of large-scale polymorphism datasets, is becoming increasingly practical. A greater reliance on McDonald-Kreitman approaches, however, calls for further investigation of its possible limitations.

**Figure 4:**
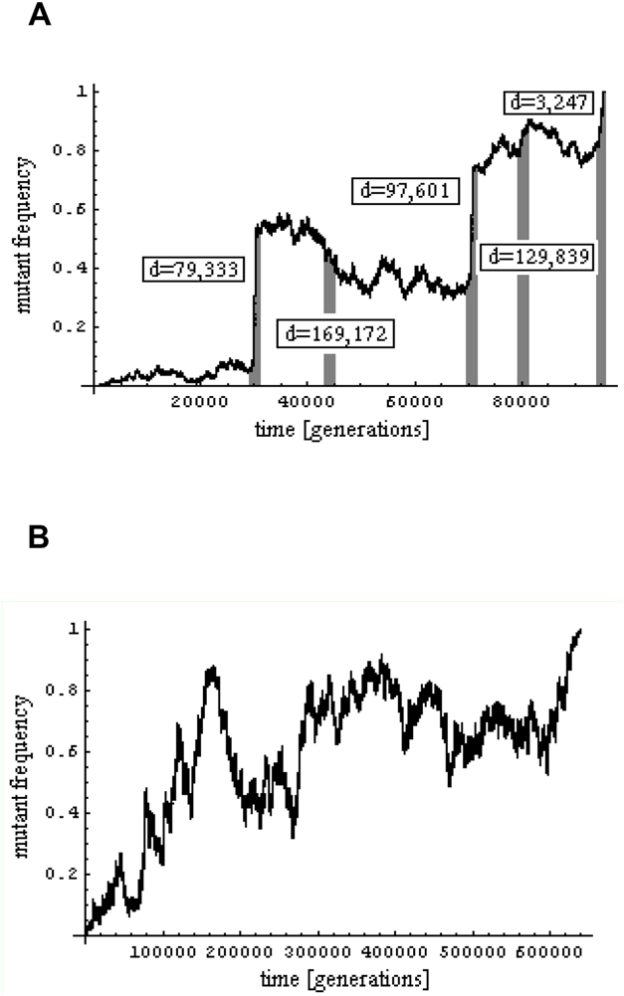
The effects of genetic draft on the trajectory of a neutral allele. (A) Simulated trajectory of a neutral allele affected by recurrent selective sweeps, from its origin on a single chromosome to fixation in the population. The population mutation and recombination parameters for this simulation are loosely based on estimates from *D. melanogaster*; the rate of adaptation, ν = 5×10^−11^, and strength of selection, *N_e_s* = 10^3^, were taken from the high end of existing estimates. The allele spent the first ∼30,000 generations drifting around low frequencies (<5%). Then, at approximately the 30,000th generation, it increased sharply and rapidly in frequency (to ∼55%) because of linkage to a strongly advantageous mutation located approximately 80 kb away; it did not reach fixation, because of recombination during the ascent of the favored allele. Subsequent to this first, dramatic change in frequency, the mutant allele experienced three hitchhiking events that increased its frequency (selective sweeps 3 through 5) and one that decreased it (sweep 2). In (B) is a simulated trajectory of a neutral allele affected solely by genetic drift, for the same population parameters. Note the difference in the time scale of the two plots.

The evidence for recurrent selective sweeps may also change our view of the population dynamics of neutral and weakly selected alleles in *Drosophila*. Figure 4 depicts a simulated trajectory of a neutral allele under recurrent selective sweeps. As can be seen, recurrent selective sweeps generate intermittent, sharp changes in the frequency of neutral alleles relative to what is expected under genetic drift alone. Thus, frequent sweeps introduce an additional and possibly important stochasticity into the dynamics of neutral and weakly selected alleles, which Gillespie termed “genetic draft” [60],[93].

Genetic draft would not affect the rate of fixation of neutral alleles—the rate of neutral evolution would still be equal to the rate of neutral mutation [94]—but it would have a bearing on many other predictions of the Neutral Theory. Relative to the expectations of the Neutral Theory, recurrent selective sweeps alter both diversity levels and allele frequencies [57]–[59]. Consistent with this prediction, a genome-wide skew toward rare polymorphisms is seen in many of the *Drosophila* species examined to date [33], [37], [38], [40], [95]–[98], and it appears to be somewhat more pronounced in regions of low recombination, at least in *D. melanogaster* (Figure 2C). Moreover, the sporadic nature of selective sweeps would cause neutral polymorphism levels along the genome to vary much more dramatically than under genetic drift alone [35],[60],[82],[89],[92]. This added variability could greatly complicate demographic inference in population genetics and ecology. The increased stochasticity would also reduce the efficacy of selection [99],[100]: while under the Neutral Theory, only alleles that are nearly neutral contribute to polymorphism and divergence, under recurrent selective sweeps, the range could expand substantially. In summary, should strong selective sweeps be common, much of the existing machinery of molecular evolution and population genetics—which is increasingly applied in the analysis of genomic data—may need to be revisited. The extent to which the current approaches are problematic depends on the rate and selective intensity of adaptations, about which little is known.

If beneficial substitutions are indeed prevalent in *Drosophila*, what are these adaptations? At present, we know too little to offer more than speculation. Evolutionary theory predicts an accelerated substitution rate associated with arms races, notably those driven by sexual antagonism and host–pathogen interactions, as well as in cases of meiotic drive [19]. Consistent with this hypothesis, an enrichment of signals for positive selection has been reported in genes with sex-biased expression in *D. melanogaster*, especially male-biased expression [35],[42],[43], as well as genes that might be associated with sexual selection, cytoplasmic parasites, and intragenomic conflicts relating to gametogenesis [35]. However, the signatures of positive selection in polymorphism and divergence are found throughout the *Drosophila* genome, suggesting that the adaptive substitutions are not restricted to a small subset of genes [32],[33]. This may point to a role of environmental shifts that drive beneficial substitutions in substantial portions of the genome. For example, changes in temperature could affect the performance of many proteins, irrespective of their function. Clearly, a better understanding of the selective pressures in *Drosophila* awaits a better characterization of these adaptations.

Insights will also be gained by studying other taxa. In this respect, we note the publication of a recent perspective [101], which focused on the work of Begun et al. in *D. simulans* [35]. It concluded that “increasing amounts of data are showing that these [the Neutral Theory's] claims and their attendant predictions do not hold for the vast majority of genes and species” (page 255 in [101]). We would argue instead that the available evidence differs markedly in both strength and clarity among organisms, and that these differences are of interest in themselves.

To date, in addition to *Drosophila*, the effects of natural selection on genome evolution have been studied primarily in primates, *Arabidopsis*, and yeast. These differ substantially in their genome sizes, ranging from ∼12 Mb in yeast to ∼120 Mb in *A. thaliana* and *D. melanogaster* to ∼3 Gb in humans [20]. In general accordance with the extent to which these genomes are streamlined (as measured, for example, by the proportion of coding DNA), the fraction of sites under purifying selection appears to be largest in yeast, intermediate in *Drosophila* and *Arabidopsis* and much lower in primates. A closer inspection, however, reveals that the fraction of coding DNA only partially predicts the levels of evolutionary constraint in the genome. For example, while *Arabidopsis* and *Drosophila* have comparable genome sizes, with a greater fraction of coding DNA in *Arabidopsis*, levels of evolutionary constraint in noncoding regions appear to be much lower in *Arabidopsis* than in *Drosophila* [102]. The explanation could lay partially with differences in population structure and effective population size [102]. The hypothesis that the effective population size largely determines levels of evolutionary constraint is strongly supported in the case of proteins, as estimates of constraint in proteins are strongly correlated with estimates of the effective population size across species [102].

The effective population size may also shape how the rate of adaptive substitutions varies among species. Under a strong selection regime, the rate will depend only weakly on population size [60], and an adaptive response may occur shortly after an environmental change [10]. In contrast, if beneficial alleles are only weakly favored, then their fixation in small populations will be impeded by genetic drift, and beneficial alleles may spend long enough in the population for environment shifts to occur before they reach fixation [10]. Among the few taxa that have been examined in depth, *Drosophila* shows the clearest evidence of extensive adaptation at the molecular level. In humans, McDonald-Kreitman-based estimates of the fraction of adaptive amino acid substitutions hover around 10% [23],[53],[84],[87],[103],[104]. Relationships of diversity with recombination [105]–[109] and of diversity with functional divergence [109] have also been detected in humans, although they appear to be weaker than in *Drosophila*. Moreover, it is harder to establish that these relationships mainly reflect the effects of selection, due to numerous confounding factors. While the finding of fewer adaptations in humans is consistent with the smaller effective population size relative to *Drosophila* species, the evidence from *Arabidopsis* and yeast is not. Both *A. thaliana* and *Saccharomyces cerevisiae*, for example, appear to have effective population sizes an order of magnitude or two larger than that of humans [110],[111], yet both show little evidence for adaptive protein evolution by McDonald-Kreitman-based approaches [112]–[115] or for the relationship between diversity levels and recombination rates [112],[116],[117]. While it is tempting to speculate that this discrepancy reflects an effect of inbreeding leading to the decreased efficacy of positive selection [102],[112],[113], we need more data points in order to make educated guesses about the causes of differences among species.

## Outlook

Although the recent findings in *Drosophila* herald a shift in our view of genome evolution, they do not yet suggest a coherent alternative picture. Among issues to be resolved, estimates of the beneficial substitution rate based on the McDonald-Kreitman methodology are considerably higher than those inferred from the relationship between polymorphism and functional divergence. This discrepancy could reflect statistical limitations of current methods, or modes of selection that have distinct effects on the two estimation approaches. For example, selection on standing variation rather than new mutations could contribute to divergence but leave little signature in polymorphism data [118]–[120], potentially leading to higher McDonald-Kreitman-based estimates. A second problem is that estimates of the selection intensity based on different methodologies differ by several orders of magnitude (Table 2). An additional difficulty lies in distinguishing the relative contributions of recurrent selective sweeps and background selection to diversity patterns.

Moving toward more reliable estimates of selective parameters will further call for the joint consideration of demographic and selective processes. Demographic events influence the dynamics of selected alleles, affecting inferences about selective parameters [121]–[124]. For example, changes in the effective population size will alter the fraction of newly arising mutations that fall within the range of weak selection (i.e., *f*) [49],[95],[121],[125]. Yet estimates based on the McDonald-Kreitman approach rely on estimates of *f* from polymorphism data—which reflect only relatively recent population history (i.e., the past ∼4*N*_e_ generations)—as a proxy for *f* over the time scale of species divergence. Under plausible demographic scenarios, this assumption can be problematic, leading to biased parameter estimates (e.g., [55]). Demographic processes can also affect inferences based on the relationships between diversity, recombination and functional divergence. Although they are highly unlikely to generate these relationships, they can distort patterns of polymorphism along the genome (for example, increasing heterogeneity in diversity levels after a population bottleneck) and, in so doing, invalidate naïve inferential models.

So where to go from here? On the experimental front, we should head toward whole-genome polymorphism and divergence data from a variety of *Drosophila* species, preferably with a range of demographic histories (e.g., endemic versus cosmopolitan species, island versus continental species). We would also gain from better estimates of basic population parameters such as mutation and recombination rates, and a more complete functional annotation of the *Drosophila* genome. On the theoretical front, we need a better understanding of different modes of selection. We also require reliable methods to infer the strength and rate of selection; as we have argued, spatial patterns of variation along the genome may be particularly informative in this respect. To gain confidence in the estimates, we will need to assess their robustness to demographic assumptions, compare estimates based on different signatures of selection, as well as rigorously test the fit of the estimated parameters to data. The resolution of these problems presents a major challenge for future research—all the more so as our understanding of molecular evolution stems primarily from inference, as opposed to direct observation. But with the development of a new generation of population genetic models and tools, and forthcoming genome-wide polymorphism datasets, it may not be long before we possess a cogent picture of the role of selection in *Drosophila* genome evolution, as well as in other taxa.
